# What do autistic children who are interested in letters and numbers do with them? A qualitative study

**DOI:** 10.1080/17482631.2025.2500851

**Published:** 2025-05-04

**Authors:** Alexia Ostrolenk, Mélanie Boisvert, Laurent Mottron

**Affiliations:** a Centre de Recherche, Évaluation et Intervention en Autisme (CRÉIA), Rivière-des-Prairies Hospital, CIUSSS du Nord-de-l’île-de-Montréal, Montreal, Canada; b Autism Alliance of Canada, North York, Canada; c St. Michael’s Hospital, Unity Health Toronto, Toronto, Canada; d Département de Psychiatrie et d’Addictologie, Université de Montréal, Montreal, Canada

**Keywords:** Autism, hyperlexia, reading, language, letters, literacy, numbers, intense interests

## Abstract

**Purpose:**

Over a third of autistic children exhibit an intense or exclusive interest in letters and numbers at the time of diagnosis. This article aims to qualitatively investigate the atypical manifestations of this interest in autism compared to typically developing children and determine if and how it can benefit children and their families.

**Methods:**

The participants were the parents of 138 autistic children (84% were non-speaking or minimally speaking) and 76 typically developing children ages 2–6. They were administered a questionnaire on their child’s interest in letters and numbers, the manifestations of these interests, the parental attitude towards it, and the child’s oral language. An inductive thematic analysis was performed on the qualitative data to establish recurring themes.

**Results:**

Eight themes were identified: atypical behaviours related to written material, emotional attachment to letters and numbers, language acquisition, use of screens, solitary behaviour, reduction of the interest over time, parental attitudes, and other special abilities.

**Conclusion:**

This study reveals that the interest in written material manifests itself in atypical ways in autism and is not comparable to the development of an interest in reading in a typically developing context. This interest also presents multiple beneficial aspects for children and their families.

## Introduction

The DSM-5 (American Psychiatric Association, 2013) diagnostic criteria for autism include “highly restricted, fixated interests that are abnormal in intensity or focus” that “interfere with daily functioning”, although this is not clearly defined. Intense interests are found in up to 88% of autistic people (Klin et al., 2007) and most individuals have more than one (Grove et al., 2018; K. P. Nowell et al., 2021). Typically developing (TD) children also have intense interests (DeLoache et al., 2007). However, autistics tend to be interested in non-social topics, while TD children’s interests are more focused on social topics and activities, considered more age-appropriate and socially acceptable (Anthony et al., 2013; Baron-Cohen & Wheelwright, 1999; Morrison et al., 2018; S. W. Nowell et al., 2019; Turner-Brown et al., 2011).

Intense interests have been tied to exceptional skills in autism. Evidence indicates that they can facilitate learning and skill acquisition in specific domains and contribute to the emergence of special abilities (Mottron et al., 2013; Happé & Vital, 2009; [Author(s)] F). Several studies have highlighted the large proportion of autistic individuals who present exceptional skills (defined as strengths compared to the general population) and personal strengths (defined as areas of high performance compared to the individual’s general level of functioning). In a large sample of autistic participants of all ages, Meilleur et al. (2015) reported that over 60% of them had at least one exceptional skill as defined in the Autism Diagnostic Interview-revised (Lord et al., 1994), and 72% of these had more than one. Exceptional memory was the most frequently reported skill. A study using parental reports in an even larger sample of autistic children and adolescents found that almost half of them had at least one exceptional skill in the areas of memory, reading, and computation, and 23% more had a personal strength. Reading as an exceptional skill was reported in 15.5% of the population, making it the second most reported exceptional talent after memory, and both domains overlapped (Bal et al., 2022). Another recent article found similar results, with at least one exceptional skill reported by a parent in 53% of their sample of autistic children (Clark et al., 2023). This widespread profile in the autistic population indicates that there may be something linking autism with exceptional skills, and this may be mediated by intense interests.

Hyperlexia, an early interest in written material together with the self-directed acquisition of reading skills and a discrepancy between decoding and comprehension, is an example of how an intense interest can lead to skill acquisition. The high prevalence of hyperlexia in autism (up to 20%) positions it as one of the most frequent special abilities in autism (Ostrolenk et al., 2017). A recently developed theory states that hyperlexia may represent an alternative path towards language acquisition in autism, using non-social linguistic material such as written text towards the eventual development of oral language abilities (Mottron et al., 2021; Ostrolenk et al., 2023). Beyond the strict definition of hyperlexia, a large-scale study led by our team revealed that over a third of autistic children exhibit an intense or exclusive interest in letters and numbers at the time of diagnosis (Ostrolenk et al., 2024). Qualitative data was also collected during the study but not included in the first article, revealing striking anecdotes reported by the parents of autistic children, including particularly unusual behaviours related to their child’s interests, which did not happen with non-autistic participants.

Previous qualitative studies on hyperlexia (see Ostrolenk et al., 2017 for a review, and Ostrolenk et al., 2023 for a recent case study) generally included a few participants and did not focus on the intense interest in letters and/or numbers in the absence of extraordinary skills. In this article, focused on qualifying atypical behaviours related to written material rather than on exceptional performance, we analyse the qualitative data collected from the parents of autistic and typically developing children on their interest in written material and identify the most recurring themes that emerged. This large-scale study provides a unique opportunity to describe a common but under-researched phenomenon through the perspectives of more than 100 participants using a rigorous methodology, since some research questions can only be answered by qualitative approaches (van Schalkwyk & Dewinter, 2020). We were particularly interested in understanding the atypical manifestations of this interest in autism, and how this interest can benefit children and their families. If this early interest is a precursor to hyperlexia and the indicator of an alternative developmental path to language and communication, it is essential that it can be described and recognized for appropriate intervention and educational practices to be developed.

## Materials and methods

### Participants

The participants of this study are the parents (87% mothers) of the groups of autistic and typically developing children included in a previous quantitative article (Ostrolenk et al., 2024, study 2). One hundred thirty-eight parents of autistic children and 76 parents of typically developing children were included in the study (Table 1). The autistic children are a subset of those referred for an autism diagnosis assessment at the CIUSSS-NIM Autism Assessment Clinic (CETSA) in Montreal, Canada over a period of 4 years, whose parents consented to participate in the study. They were all formally diagnosed with Autism Spectrum Disorder according to DSM-5 criteria (American Psychiatric Association, 2013) following an in-depth interview between the parents and one or two clinicians and the administration of the Autism Diagnostic Observation Schedule (ADOS-2; Lord et al., 2012) to all but 5 children. The CETSA conducts assessments in French. The TD group was recruited from six daycares within the same geographical area, and children were required to have no first-degree relatives with neurodevelopmental conditions. Information on race/ethnicity and socioeconomic status were not collected.

**Table 1.** Participants demographic information.

	Autistic group	Typically developing group
	N = 138	N = 76
Sex, n (%)		
Female	32 (23)	36 (47)
Male	106 (77)	40 (53)
Age (months)*		
Mean	51.31	47.54
SD	11.72	15.74
Range	24–80	24–83
ADOS, n (%)		
Toddler module	4 (3)	N.A.
Module 1	112 (81)	N.A.
Module 2	16 (12)	N.A.
Module 3	1 (1)	N.A.
Not assessed with an ADOS	5 (4)	76 (100)

*There was no significant difference between the ages of the groups.

### Materials

A caregiver questionnaire was developed for the purpose of this study, the *Questionnaire sur l’intérêt pour le matériel écrit chez les tout-petits* (QIMET). It consists of a 45-minute parental semi-structured interview conducted over the phone including open-ended and close-ended questions. The questions cover the child’s interest in letters and numbers, the manifestations of these interests, the parental attitude towards it, and the child’s oral language skills. Immediately after the phone interview, the evaluator records a 5-point quality score based on the information collected during the questionnaire through answering 3 questions about the precision of the responses, the parental knowledge of the child’s behaviour, and their general impression on the validity of the reported information. The evaluator could also write comments to justify their score (e.g., sound quality, level of distraction or impatience of the parent). Participants with a quality score lower than 2 out of 5 were not included in the analysis.

### Procedure

All the parents of children under 7 years old assessed at the CETSA over a period of 4 years were contacted about this study in the days following the assessment. Inclusion criteria were to be able to answer a questionnaire in French and to have known the child since the age of one. If a parent agreed to participate, a phone appointment was scheduled within a month following their child’s assessment for them to complete the caregiver questionnaire (QIMET). Parents of TD children were recruited directly through their daycare. The questionnaire was conducted and recorded by trained female graduate students and research assistants, including one of the authors (MB). A total of 341 interviews were conducted in the context of a large-scale mixed-methods study. The previously published quantitative study included one autistic group (*N* = 138), one non-autistic clinical group (*N* = 99), and one typically developing group (*N* = 76), but the present one focuses solely on the differences between the autistic and TD groups. Participants were included in the analysis for this study if they had received a diagnosis of autism or were TD (excluding the 99 non-autistic clinical children), if their data was complete and their quality score on the QIMET was above 2/5 (26 participants excluded), resulting in a total of 214 included participants. Additional information on the autistic group was collected from the clinical files, including the ADOS module chosen to assess the child, which reflects their level of oral language (the Toddler module is recommended for children 12–30 months who say no words or some isolated words; module 1 is recommended for children over 31 months who say no words or some isolated words; module 2 is recommended for children who can make short sentences at most; module 3 is recommended for children with fluent language). 84% of the autistic group had minimal oral language abilities, as indicated by the use of ADOS Toddler module or module 1.

### Analysis

We used the notes and audio recording taken by the interviewers during the questionnaire to transcribe the parents’ answers. Open questions included: “How did you notice that your child had an interest in letters/numbers that was stronger or more precocious than other children?”, “How do you think that your child developed this interest and these skills related to reading, writing, and written material?”, “Does your child have other abilities that surprise you?”, and “Is there any other thing of interest that you would like to mention about your child?”. An inductive thematic analysis (Braun & Clarke, 2006) was performed using the guiding principles of Srivastava and Hopwood’s (2009) framework. The research team, all familiar with the data, first established a list of recurring themes that were noticed during the data collection and curation. Additional themes were added as needed when going through the qualitative data if a recurring theme emerged that we had not identified before, or if a particularly striking difference between both groups was observed. MB and AO then systematically reviewed the data for all participants to classify each statement into a main theme, and a secondary theme if relevant. The final names and definitions for the themes were developed throughout this process.

To investigate non-speaking autistic children, we created a subgroup whose parents reported that they did not communicate using spoken words at all. Those who used echolalia or made-up words were included in the non-speaking subgroup. All TD children had appropriate oral language skills for their age as reported by their parents. The Consolidated criteria for reporting Qualitative research (COREQ; Tong et al., 2007) 32-item checklist for interviews was used to ensure the quality of reporting (see Supplementary Materials).

### Ethics statement

This research project was approved by the CIUSSS-NIM research ethics committee (study number 2019–1793), and all participants gave informed consent.

## Results

Eight themes were identified. The list of themes along with a short description of each are presented in Table 2. All the quotes included below have been translated from French to English. When the TD group is not mentioned in comparison to the autistic group, it means that the behaviour was not reported in any of the TD children.

**Table 2.** List of themes used to classify the qualitative data.

Theme	Description
1- Atypical behaviors related to written material	Anecdotes related to the child’s spontaneous behavior with letters/numbers that were atypical, showing the strength and compulsive nature of the interest.
2- Emotional attachment	Evidence of emotional attachment to written material and its soothing purposes.
3- Language acquisition	Unusual modes of acquisition of language skills and foreign alphabets. Vocalization of letters and numbers in non-speaking children.
4- Use of screens	Use of screens (computer, phone, tablet, television) to develop skills related to letters/numbers.
5- Solitary behavior	Description of literacy-related activities as self-directed or solitary, refusal to share these activities with an adult.
6- Reduction of the interest over time	Decrease of a previously very strong interest in letters/numbers.
7- Other special abilits	Other special abilities mentioned by the parents.
8- Parental attitude toward reading	Parental attitude towards reading and the effect on the child’s behaviors associated with written material.

Of the 138 autistic children, 33 were identified as non-speaking if their parents reported that they did not communicate using spoken words at all. All of them were assessed using ADOS Module 1 or Toddler module. 18% of the non-speaking children had an intense or exclusive interest in letters and numbers, 27% of them could count, 18% would sing the alphabet song, and 24% would name letters. Three non-speaking children could write letters and numbers, two could recognize some written words and one could read. Notably, 30% of parents mentioned their child’s intelligence, with statements such as “he is intelligent despite being non-speaking” or highlighting incredible abilities in executive functioning tasks.

### Atypical behaviors related to written material

At age 3, he was already looking at his sister’s books in grade 4 […] and was interested in dictionaries. […] he hardly spoke at all, but he could write. […] Now he’s interested in 5th and 6th grade French and vocabulary books. - Mother of a 5y 1m autistic son. (ID #112)

Parents were often surprised by how rapidly their child acquired new skills related to reading and writing, and three parents of autistic children reported that they discovered skills when they were already mastered. Six parents of autistic children mentioned the use of anything and everything to form letters and numbers: play dough, food, dirt, even saliva if nothing else is available. An autistic child collected packaging from trash cans in the park just to look at the writing on it, another counted everything, and 13 autistic children had to name letters and numbers whenever they saw them in their environment. No such behaviours were found in the TD group.

When asked about the first time they understood that their child was interested in letters or numbers, a parent told us an anecdote from when their autistic child was 12 months old: his grandmother dropped a newspaper (that did not contain images) by accident, the child picked it up, turned it so that it would be in the right orientation to read, and refused to let go of it, starring at the written text. In some cases, the interest in letters mixed with the interest in numbers: one autistic child often counted the number of letters in a word, and another was fond of licence plates. Five of the autistic children who were strongly interested in letters did not particularly like books or images, as opposed to TD children.

### Emotional attachment

He turns everything he finds into an alphabet. […] He liked it so much, Madame, that he slept with the alphabet in his bed. - Mother of a 5y 1m autistic son. (ID #112)

Several children showed signs of emotional attachment to letters and numbers. One child slept with foam letters of the alphabet, another always wanted his favourite book under his pillow, and another needed to have multiple books in his bed with him. Two autistic children were particularly attached to a random specific letter. This phenomenon was also found in two TD children, but it was the first letter of the children’s name. Nine parents of autistic children mentioned smiling and joy related to letters and numbers, and 3 said they used them to calm their child when needed, for example by singing the alphabet.

### Language acquisition

His first words were letters and numbers. - Mother of a 3y 3m autistic son. (ID #094)

Fourteen autistic children’s first spoken word had been the name of a letter or a word they had read. Ten autistic children’s parents reported the spontaneous emergence of reading skills without prior instruction which they attributed to passive exposure, and one parent of a TD child. A mother was surprised by her autistic son’s vocabulary when he pronounced complex words that she never used. Another parent said that her autistic child “talked like a book” because he used overly formal language. Interestingly, 51.3% of the autistic children presented with delayed echolalia, but this number rose to 67.3% in those with an intense or exclusive interest in letters.

I don’t know why […] he prefers English, even though he was born in an Arabic/French-speaking environment. We don’t speak English. - Mother of a 4y autistic son. (ID #071)

Several cases of “unexpected bilingualism” were identified. A quarter of the autistic children in our sample, as opposed to 5% of TD children, could name letters in a language that was not spoken at home or at daycare/school. In autism, there were over 20 mentions of a strong interest in foreign languages and foreign alphabets, and only one mention in a TD child. Twelve autistic children had learnt some English independently although their parents did not speak it. The independent use of Spanish was also mentioned more than once, and so was the learning of foreign alphabets such as Russian through the television or the use of a tablet. In several cases, autistic children showed a strong preference for English, sometimes to the point of refusing to speak the language most present in their environment (usually French), even when the parents could not understand English. For example, an autistic girl had spontaneously started speaking English at 18 months and went through a phase around 30 months when she stopped speaking French although it was her native language. She learnt letters and numbers in English through songs. She sometimes mixed both languages, for example when counting down the seconds left on the microwave saying “twenty-trois, twenty-deux, twenty-un”. Another autistic child, who also started speaking English before French and used it most of the time although her parents and brother did not speak it, would use a tablet to listen to the alphabet song in languages that her parents could not even identify. One child sang the alphabet and counted in Spanish, German, and Mandarin, although only French and English were spoken around him. Parents often made the hypothesis that the source material for foreign language acquisition came from movies, videos, and games. In some cases, though, they had no idea where it came from.

He counts in English, French and Spanish, well Spanish, I don’t understand Spanish, the babysitter told me he counted in Spanish, but I didn’t know he counted in Spanish […] I don’t understand Spanish. - Mother of a 3y 2m non-speaking autistic son. (ID #038)

### Use of screens

I didn’t teach her English, she learned it on her own with nursery rhymes […] at 18 months. - Mother of 3y 11m autistic daughter. (ID #063)

Screens played an important role in the autonomous acquisition of linguistic skills. The onset of the interest was often associated with watching a show or YouTube videos that introduced letters or numbers. One parent said that their autistic child was interested in playing with a tablet only if the game related to the alphabet. Another child, aged 5 years and 9 months, would use a search engine by herself to find out how to spell new words that she was curious about. Although some parents were reluctant to let their child use screens for extended periods of time, 7 parents of autistic children mentioned that they were a precious help to nourish their child’s interest and foster the acquisition of new skills.

Every week he learns [how to write] 5 or 6 new words […] with the Foufou channel program, if it’s something he’s interested in […] he pauses, writes the word on his board and it sticks to his memory. - Mother of a 5y 1m autistic son. (ID #112)

### Solitary behaviour

Many parents did not know how their child had learnt to read and said that the interest was fully self-directed and the skills self-taught, at least at first. Autistic children often did not like an adult intruding into their activities, and rarely enjoyed shared reading, with four children refusing categorically, which was never the case in TD children. A non-verbal child aged 2 years and 11 months who had learned letters and numbers in French and English by herself would refuse any learning activity accompanied by an adult and refused to show her skills when asked. One boy would cover his ears or cover his mother’s mouth every time she tried to read him a book. Another child who had an exclusive interest in letters and numbers and had learnt the alphabet and numbers by himself in 6 languages (English, French, Spanish, Italian, Arabic, and some Chinese characters), would get angry if anyone touched his toys or books. The opposite profile was present in some of the TD children: they would not read or write without an adult encouraging them to do so.

### Reduction of the interest over time

Twelve parents of autistic children noted the reduction of the interest in letters/numbers when it used to be very strong. The interest often peaked around age 2, and the reduction appeared around age 4, drastically in more than one case. The reduction was also generally associated with the development of new interests, suggesting that the interest may have expanded to other themes once the child had fully explored it, rather than disappeared. This reduction in interest was not present in the TD group.

### Other special abilities

When questioned about other special abilities they may have noticed in their child, many parents mentioned that they were amazed by their child’s memory, especially for text and numbers. We found that memory was almost never mentioned in children with no interest in letters and was almost always mentioned in participants with an exclusive interest in letters, in both autistic and TD participants. Overall, 40% of parents of autistic children and 42% of parents of TD children who had an intense or exclusive interest in letters spontaneously mentioned that they had an exceptional memory. In the autistic group, other special abilities related to visual perception were often mentioned (drawing, construction, puzzles, visual memorization, orientation in space, interest in colours and shapes) as well as musical abilities.

### Parental attitude

For us, [listening to letter programs], it was the way to develop his language and knowledge of words and sentences. […] We thought maybe this activity would help him speak better, express himself better. […] We encouraged him except for one small restriction: we didn’t want it to be excessive, we wanted him to be interested in something else. - Mother of 4y 4m autistic son. (ID #054)

To our question “What is your attitude towards reading”, five parents of autistic children (and none in the TD group) responded “reluctant”; four of these children were non-speaking or minimally speaking (used no words or only isolated words to communicate). The main reason for the parents’ reluctance was that the interest was too intense and restrictive. The mother of a 3y and 6m non-speaking autistic son (ID #101) who devoted 100% of his free time to letters and numbers felt that the interest was too intense and that her son should learn to communicate verbally before focusing on letters and numbers, so she decided to prevent her son from engaging with his interest. Another mother of a 3y and 6m minimally speaking autistic son (ID #140) with an intense interest in letters and numbers reported that she prevented him from watching letter- or number-related programmes and limited his interest in written material. A health professional had told her that it was not in her child’s best interest to be so focused on letters and numbers when he had not achieved autonomy and his speech was delayed. In contrast, two caregivers had been encouraged by clinical professionals to introduce their autistic child to written material to stimulate their language abilities. They reported that their children’s verbal skills had improved as a consequence, although the causal relationship could not be established with certainty.

## Discussion

This study aimed to identify the qualitative differences between autistic and TD children in the way they manifest their interest in letters and numbers. Our findings demonstrate the presence of unique behaviours related to letters and numbers in the autistic group that were not present in the TD group. This robust qualitative study is the first of its kind on this topic, including an in-depth interview with a large sample of participants and a matched comparison group, which is often missing in qualitative research (Lindsay, 2019).

### Atypical manifestations of the interest

The interest in written material found in autistic children is of a different nature and manifests itself in different ways than in TD children. First, the developmental path of reading acquisition is different: autistic children with an interest in letters and/or numbers were often minimally speaking, and their early vocabulary contained essentially the names of letters and numbers. In several cases, the first word uttered by a child had been read, and new vocabulary was acquired essentially through reading (that was also the case for a pair of hyperlexic and autistic twin brothers investigated in a case study; Ostrolenk et al., 2023). In contrast, TD children’s language skills were age-appropriate and already well developed when the interest appeared. They were rarely interested specifically in letters, but more in books and shared reading. Conversely, autistic children engaged in literacy-related activities by themselves and often did not accept external intervention. Our previously published quantitative results showed that autistic children predominantly engaged in non-social behaviours related to their interest in letters, such as aligning letters and looking at books alone. On the other hand, TD children engaged in more social behaviours, such as reading for others or bringing a book to be read by an adult (Ostrolenk et al., 2024). Autistic children’s skills were self-taught and their ability to acquire new skills, including new languages, without explicit instruction or social interaction was astounding and never observed in a TD population. This phenomenon, labelled “unexpected bilingualism”, has been reported previously (Kissine et al., 2019; Vulchanova et al., 2012; Zhukova et al., 2021) and included in a proposed list of signs of prototypical autism (Mottron & Gagnon, 2023). Moreover, the use of screens as a support for learning was overwhelmingly present. Certain compulsive behaviours related to the children’s interest in letters were unique to the autistic group and never reported in TD children, so were signs of emotional attachment to letters and numbers.

The finding that autistic children may refuse shared activities related to reading in spite of having a solitary interest in them is not unique to our work. For example, Gina, a 15 year-old who had learned to read independently at 3 years old was “not interested, and often resistant, to reading books with her parents” (Robertson, 2019), and RH, a hyperlexic toddler who could place letters in alphabetical order and spell his name with foam letters before age 2, but “resisted having books read to him” (Gernsbacher, 2004). In a study collecting the experiences of parents of autistic children regarding home reading, Walker et al. (2022) mentioned that engaging the children in shared reading activities could be challenging. Lanter et al. (2013) also reported a discrepancy between the level of lone enjoyment of letters and shared activities related to reading in autistic children compared to language-matched peers. These are only a few examples of what seems to be a widespread profile in autism. Shared book reading is a highly social activity that usually requires joint attention and reciprocal interactions between the child and the adult. The atypical development of socio-communicative skills in autism even before the age of diagnosis may have an impact on the frequency and quality of shared book reading from a young age. A low level of enjoyment and interaction by the child may result in the parents spending less time on this activity.

### Relationship with language

Our autistic group was mostly composed of minimally speaking autistic children who are just over 4 years old on average, making their interest in written material even more surprising. A subgroup of 33 of them were considered non-speaking by their parents. Although they did not use words to communicate, many of them were interested in letters and numbers and several could count or recite the alphabet. These 33 non-speaking children may represent a developmental stage that other children in our sample have passed through before their oral language emerged.

We also found that echolalia was more frequent in autistic children with an intense or exclusive interest in letters than in those without. Echolalia is often observed in autistic children with hyperlexic traits (Cardoso-Martins et al., 2013; Craig & Telfer, 2005; Lin, 2014; Solazzo et al., 2021). Although echolalia was previously believed to be non-functional, there is evidence that it plays a role in speech acquisition, and experts recommend that intervention strategies should not target its elimination (Stiegler, 2015). The hypothesis that hyperlexia could be the “written language analogue” of echolalia has been proposed as early as 1975 (as reported in Cobrinik, 1982). The frequent presence of echolalia in children with hyperlexic features is another indication of future language emergence.

### Loss of interest over time

Several parents of autistics noted that their child’s interest in letters and/or numbers peaked around 2 years old and reduced, sometimes drastically, after the age of 4. Although this question is seldom explored, other studies have also reported this phenomenon (Sparks, 1995, 2001; Talero-Gutierrez, 2006). There are several potential explanations for this. First, there is a virtual limit to how much can be learnt about letters and a ceiling on decoding ability. Eventually, children may master letter-related skills and move on to more challenging interests. It is also possible that parents may report a reduction of the specific interest in letters when their child’s fields of interest expand, when it is still present but less obvious. The reduction of the interest in letters may also be associated with the improvement of social skills and acquisition of oral language. Researchers have hypothesized that hyperlexia was an alternative learning path towards language, mediated by written information in the first stages, which could lead to the eventual development of oral language abilities (Mottron et al., 2021; Ostrolenk et al., 2024). It is possible that once this is done, the child does not need written language as much anymore.

Moreover, we have shown that autistic children preferred practicing their literacy skills independently from adults. Around the age of 4, pre-literacy teaching becomes more important, and children are explicitly encouraged to acquire reading and writing skills. It is possible that this has a negative impact on the autistic children’s interest in these activities. There may also be a disconnect between the skills that early reading instruction usually focuses on and the skills that hyperlexic autistic children are practicing (Macdonald et al., 2022): adults may be prone to teaching a 5-year old the basis of single word decoding, when this skill is already fully acquired by a hyperlexic child who would rather engage in a more novel exercise. Furthermore, formal instruction usually involves social interactions in a classroom context and mandatory reading material. When autistic children reach the typical age for formal reading instruction, a lot of them are already reading (as in, decoding) far above grade level and may be bored with the materials proposed. In a qualitative study about autistic adults’ reading habits, several reported that forced educational reading demands had reduced their enjoyment of reading (Chapple et al., 2021). In our work, we have found that autistic children were sometimes inflexible regarding their activities of interest and did not appreciate when adults interfered. The disinterest may thus result from misguided support for the development of autistic children’s reading skills (Newman et al., 2007).

### Relationship between intense interests, intelligence, and other exceptional abilities

Unusually good memory was spontaneously mentioned by 40% of the parents of children who had an intense or exclusive interest in letters, along with other perception-related strengths in autism. This intense interest is the first sign of hyperlexia, which has been associated with other exceptional abilities in autism, such as strong memory skills (Nation, 1999, Sparks, 1995) and perceptual strengths (Turkeltaub et al., 2004). Savant autistics tend to have superior working memory and pattern perception abilities (Bennett & Heaton, 2017; Mottron et al., 2009). Exceptional domain-specific abilities are very frequent in autism, memory being the most frequently reported, showing an overlap with exceptional reading skills (Bal et al., 2022; Meilleur et al., 2015). Common cognitive characteristics—such as a strong memory—may explain various skills, and hyperlexia may emerge for the same reasons that other exceptional abilities emerge in autism. Intense interests can lead to a quasi-exclusive dedication of time and cognitive resources to the material of interest that facilitates skill acquisition in specific domains, thus contributing to the emergence of special abilities (Happé & Vital, 2009; Mottron et al., 2013). A double rededication, both behavioural and functional, could explain how a precocious early interest in letters may develop into fully recognizable hyperlexia. More generally, many parents made a point of talking about their child’s intelligence despite their lack of oral language. Too often, the inability to speak is misconstrued as a lack of willingness to communicate or equated with a lack of intelligence. As demonstrated here, children with minimal verbal skills may display highly intelligent behaviours and intent to communicate, albeit in unconventional ways.

### Parental and clinical attitudes regarding intense interests

Some parents of autistic children hold reluctant attitudes regarding their children’s interest in letters and numbers. One of them had been advised by a clinician to prevent her child from engaging with letters and numbers and focus on his oral language instead. This is not the case for TD children, for whom literacy-related activities are generally well perceived. Early conceptualizations of intense interests in autism focused on their pathological nature and called for their suppression in favour of more socially acceptable behaviours (Lovaas, 1981, pp. 350–351). Although we would like to think that these beliefs are outdated, they are still held by some professionals in the autism field. In a recently published handbook, the authors state that punishment could be used for behaviours that “compete with learning” (Matson, 2023). Fortunately, the recent years have seen a shift towards recognizing the positive aspects of intense interests and asking autistic people about their opinion on this matter. These investigations have shown that intense interests can be a source of wellbeing and motivation (Grove et al., 2018), lead to employment opportunities (Koenig & Williams, 2017), and better educational and social outcomes (Wood, 2019). Additionally, autistics have reported that being able to share their interests and having them be socially accepted contributed to their quality of life (Epstein et al., 2017), and research has found that a positive view of the attributes of autism led to better self-esteem in autistic people (Cooper et al., 2020). These results call for an evidence-based change of perception of intense interests in autism in favour of autistic people’s wellbeing and successful development.

### Implications of the findings

Our findings have multiple implications. As the emerging signs of hyperlexia are being uncovered, their relation with typical or autistic language acquisition is still poorly understood. Our results suggest that autistic children’s atypical interest in written material, and resulting hyperlexia, are part of a distinct developmental trajectory to language acquisition. The precocious emergence of this intense interest and related early reading skills is followed in a significant proportion of cases by a prototypically autistic plateau without visible progress in oral language, and then by a relative diminution of the interest around the age of 4, when oral language skills may progress. Oral language sometimes appears in a language other than the ones used at home, depending on the media consumed by the child. These autistic children tend to acquire skills through self-directed practice granted the material of interest is accessible, and not through explicit teaching by an adult. Hence, they acquire knowledge in a different order and through different mechanisms than their typically developing peers. Intrinsic reading motivation positively affects reading achievement (Hebbecker et al., 2019; Schiefele et al., 2012), and early literacy skills in autism predict later reading skills (Knight et al., 2018). Thus, the interest in written material and the precocious development of decoding skills that characterize hyperlexia are likely to play a role in academic and social achievement if appropriately supported. Understanding this atypical learning trajectory that can support the acquisition of reading and language skills has crucial implications for adapted education.

Although hyperlexia could have a positive impact on academic achievement, it is also associated with reading comprehension difficulties—at least in its early stages- that can have a negative impact on academic performance if left unaddressed. The unique profile and set of skills specific to hyperlexia leads to unique needs for intervention strategies that consider both cross-sectional and longitudinal autistic children’s specific profile of strengths and weaknesses. There is evidence that interests can be embedded in intervention strategies to support learning (El Zein et al., 2016; Harrop et al., 2019; Solis et al., 2022). However, when impaired children show an interest in objects directly related to literacy, such as books, it can be misinterpreted and dismissed as non important (Mirenda, 2003). Our study sheds light on a frequent intense interest in autism and the peculiarities of its manifestations. This will allow for an increased recognition of this interest in autistic children, opening the door to the incorporation of children’s strengths and interests when addressing areas of difficulty. Such strategies are a promising avenue for intervention in hyperlexia, but also for supporting families with day-to-day management and improved communication at home or at school.

Strengths and interests can also be taken into account when choosing the mode of delivery for an assessment or intervention. Many autistic children are particularly interested in technology, and screen-based activities may be more successful than human-led interactions for this population. Screens have a bad reputation in autism because of the suspected ties between screen time in infancy and the development of autism (Kushima et al., 2022). Alternatively, our results suggest that screens are often the source of information from which autistic children learn independently: most parents report that tablets and computers are responsible for their children’s self-taught skills. Denying them access to these tools would mean denying them opportunities to learn in the way that works best for them.

## Conclusion

This study adds to our understanding of the behavioural differences between autistic and TD children relative to letters and numbers. The interest in written materials manifests itself in atypical ways in autism and is not comparable to the development of an interest in reading in a typically developing context. Autistic children show more behaviours of interest that do not involve social interactions (e.g., aligning letters), while TD children favour social sharing of this interest (e.g., shared book reading). Although we use the same words to define behaviours in autistic and non-autistic children (e.g., being interested in something, reading, etc.), they may not mean the same thing for both groups. This can have practical impact when interrogating individuals who may have diverging interpretations of these words depending on their context, potentially confounding the results of a study. It also appears that this interest, when present, can be a source of well-being and lead to the acquisition of new skills. Thus, we should discard the idea that intense interests are an obstacle to the acquisition of language, and further investigate them and develop tools to identify them.

## Supplementary Material

Supplementary_QualiHL_ISSM_COREQ_Checklist.docx

## Materials availability

The data collected for the current study cannot be made publicly available due to the detailed behavioural descriptions and medical information that could allow for participants’ identification.
